# Evaluation of the transverse aortic constriction model in ICR and C57BL/6J mice

**DOI:** 10.3389/fphys.2022.1026884

**Published:** 2022-11-29

**Authors:** Mengying Huang, Lishuang Yu, Xiaoping Wang, Mingmin Wang, Weili Li, Jiayang Tang, Guanjing Ling, Xiaoqi Wei, Yong Wang, Wei Wang, Yan Wu, Linghui Lu

**Affiliations:** ^1^ School of Traditional Chinese Medicine, Beijing University of Chinese Medicine, Beijing, China; ^2^ Endocrinology Department, First Affiliated Hospital of Zhejiang Chinese Medical University, Hangzhou, China; ^3^ Beijing Key Laboratory of TCM Syndrome and Formula, Beijing, China; ^4^ Key Laboratory of Beijing University of Chinese Medicine, Ministry of Education, Beijing, China; ^5^ Research Institute of Chinese Medicine, Beijing University of Chinese Medicine, Beijing, China

**Keywords:** transverse aortic constriction, ICR mice, C57BL/6J mice, cardiac hypertrophy, heart failure, pressure overload

## Abstract

Transverse aortic constriction (TAC) is a frequently used model to investigate pressure overload-induced progressive heart failure (HF); however, there is considerable phenotypic variation among different mouse strains and even sub-strains. Moreover, less is known about the TAC model in ICR mice. Therefore, to determine the suitability of the ICR strain for TAC-induced HF research, we compared the effects of TAC on ICR and C57BL/6J mice at one, two and four weeks post-TAC *via* echocardiography, organ index, morphology, and histology. At the end of the study, behavior and gene expression patterns were assessed, and overall survival was monitored. Compared to the sham-operated mice, ICR and C57BL/6J mice displayed hypertrophic phenotypes with a significant increase in ventricle wall thickness, heart weight and ratio, and cross-sectional area of cardiomyocytes after a 2-week TAC exposure. In addition, ICR mice developed reduced systolic function and severe lung congestion 4 weeks post-TAC, whereas C57BL/6J did not. Besides, ICR mice demonstrated comparable survival, similar gene expression alteration but severer fibrotic remodeling and poor behavioral performance compared to the C57BL/6J mice. Our data demonstrated that ICR was quite sensitive to TAC-induced heart failure and can be an ideal research tool to investigate mechanisms and drug intervention for pressure overload-induced HF.

## Introduction

Heart Failure (HF) represents a global health and economic burden, with over 64 million people affected worldwide. Despite significant improvements in HF therapies, morbidity and mortality of this life-threatening syndrome remain high (Savarese et al., 2022). Hypertension remains a common and powerful risk factor for developing HF. Generally, chronic pressure overload impels left ventricle (LV) hypertrophy, progressive fibrotic alteration, and diastolic and systolic dysfunction. However, multiple manifestations and mechanisms are involved in this deteriorative progression and most remain elusive (Di Palo and Barone, 2022). Therefore, researchers continue to study the mechanism and pharmacology of pressure overload-induced HF using rodent animal models.

Transverse aortic constriction (TAC) is an experimental model frequently used to induce pressure overload on the heart, mimicking hypertensive HF in humans. First introduced by Rockman and co-workers in 1991 (Rockman et al., 1991), the TAC model has been extensively applied with continuous technical improvement. A minimally invasive transverse aortic constriction (MTAC) was introduced and widely accepted due to a major selling point: the aortic arch can be reached and constricted without thoracotomy and artificial ventilation with tracheal intubation (Zaw et al., 2017). Besides the great improvement, there is a concern about phenotypic variability response to TAC, probably depending on multiple study characteristics. Recently, a TAC model systematic review and meta-analysis indicated that the heterogeneity might be associated with a strong bias from specific mouse line preferences and other factors (Bosch et al., 2021). Therefore, studies have investigated how mouse strain affects TAC-related phenotypes. According to the meta-analysis, C57BL/6 mice are ahead of the other strains, accounting for 67% (344/472) of the studies (Bosch et al., 2021). However, three commonly used sub-strains of C57BL/6 mice displayed distinct responses to TAC stimulation. C57BL/6J gained more preference since it developed earlier than other sub-strains, and many genetically modified mouse lines were bred on this background (Garcia-Menendez et al., 2013). C57BL/6NTac or C57BL/6NCrl displayed poor survival, severe interstitial fibrosis, higher expressed hypertrophic markers, and better phenotypic homogeneity compared to C57BL/6J mice (Garcia-Menendez et al., 2013; Zi et al., 2019). Similarly, a recent study compared BALB/c and C57BL/6J mice following comparable TAC. Despite the similarity in hypertrophic remodeling, BALB/c mice progressively developed ventricle dilation, systolic dysfunction, severe lung congestion, and considerable mortality, whereas the C57BL/6J strain did not (Tannu et al., 2020). Therefore, the mouse strain should be considered when using the TAC model to explore the mechanisms of hypertensive heart failure.

MTAC simplified the surgery procedure; however, TAC’s operation difficulty and less survivability are undeniable, especially for novice investigators. Many mice would be consumed inevitably from TAC surgery initiation to a fully conducted pharmacological or genetic investigation. Considering the economic burden, ICR mice would be a better choice. Usually, the ICR price is approximately 1/4-1/3 of C57BL/6J mice in China. In addition to the cost advantage, ICR mice have larger bodies that facilitate reducing the surgical difficulty and harvest more blood and tissue samples for further molecular research. In a recently published protocol paper, a German team selected ICR mice to establish the first neonatal TAC model (nTAC) despite the exact reason being undisclosed (Malek Mohammadi et al., 2021). Additionally, ICR mice were also preferred by a Japanese lab to study the cardioprotective mechanism of σ_1_-receptor stimulation on myocardial hypertrophy induced by TAC (Tagashira et al., 2010). However, we knew less about ICR TAC models and there has yet to be a study conducted to fully evaluate the TAC model in ICR compared to the most popular C57BL/6J strain.

Thus, we performed the MTAC surgery on both ICR and C57BL/6J mice and assessed survival, cardiac structure and function, heart and lung weight ratio, morphological changes, behaviors, and gene expression profiles at one, two and 4 weeks post-TAC, hoping to provide detailed time-course parameters for future studies.

## Materials and methods

### Animal care

This study was performed on male mice of SPF-grade ICR (5 weeks old) and C57BL/6J (7–8 weeks old). All the mice were purchased from SPF (Beijing) Biotechnology Co., Ltd., China and bred at 22°C ± 2°C with the appropriate humidity and 12-h lighting/dark cycle. The mice were allowed 1 week’s acclimation preoperatively. All the mice experiment procedures were approved by the Beijing University of Chinese Medicine Animal Care (BUCM-4-2021110902-4045).

### TAC surgery

Minimally invasive TAC was performed as previously described (Zaw et al., 2017) with minor modification. Briefly, the mice were anesthetized with pentobarbital (50 mg/kg i.p.), followed by hair removal on the front neck and chest using depilatory cream. They were kept in the supine position with their heads towards the operator. The skin was open at the midline of the neck and chest, the muscle layer was separated towards both sides, and then the sternum was cut to the second rib. Carefully separate the thymus lobes and connective tissue till transverse aortic arch and two carotid arteries are clearly visible. The transverse aorta was isolated between the carotid arteries and ligated using a 6-0 nylon suture ligature tied firmly against a 27-gauge spacer. The spacer was gently removed, and the chest layer was closed by layer using a 5-0 silk suture. Mice was placed on a warm pad until anesthesia recovery that usually taking 2–3 h. The sham mice underwent a similar surgical procedure without aortic constriction, serving as controls.

### Echocardiographic analysis

Echocardiographic tests were performed when the mice were anesthetized with 1% isoflurane using VisualSonics Vevo 2100 echocardiographic system (FUJIFILM VisualSonics, Canada) equipped with a 30-MHz probe (MS-400). The transverse aortic constriction was visualized using 2-D and color flow imaging. The aortic flow velocities were acquired using pulsed-wave doppler detection to determine appropriate constriction or sham. The study’s inclusion criteria were Vel_sham_ ≤ 800 mm/s and Vel_TAC_ ≥ 2400 mm/s. From the 2-D parasternal short-axis view, an anatomic M-mode echocardiogram was recorded at the papillary muscle level. All the films were analyzed offline with VEVOlab software. Ventricular structure indicators, such as left ventricle anterior and posterior wall thickness (LVAW and LVPW, respectively), and LV internal diameter (LVID) both at end-diastole and end-systole, were directly measured. Other indicators were automatically calculated using the software. Relative wall thickness was estimated using RWT = (LVAWd + LVPWd)/LVIDd × 100% (Støylen et al., 2016). All the measurements were blindly carried out and all data acquired from three consecutive cardiac cycles for average. A group of 6–10 mice from each strain were sacrificed at 1, 2 and 4 weeks after the echocardiography for doing the other tests.

### Tissue harvest and photography

Following echocardiography, hearts and lungs were rapidly harvested and weighed. Hearts were quickly photographed with the scale. A transverse mid-LV section was immersed in 4% paraformaldehyde for at least 24 h and then embedded in paraffin. Subsequently, they were cross-sectioned into 5 μm pieces for histological staining. The left heart tissues were frozen in liquid nitrogen for RNA analysis. In addition, the right tibia length (TL) was measured after dissection. The heart shape index was calculated as previously defined (Dvornikov et al., 2019), which equals ventricle area/perimeter^2^. Those parameters were measured using ImageJ software based on the heart photos.

### Histology analysis

Slides were processed using Hematoxylin and eosin (H&E) and Masson’s trichrome staining to assess heart structure and fibrillar collagen infiltration. Wheat germ agglutinin (WGA) staining were used to evaluate cardiomyocytes contour following the manufacturer’s instructions (Servicebio). Images were captured using a Pannoramic DESK scanner (3D HISTECH). Additionally, the fibrotic area ratio and myocardial cross-sectional area were quantified using ImageJ software.

### Behavioral observation evaluation

The behavioral observations were designed by referring to the Irwin test and functional observational battery as previously described (Mathiasen and Moser, 2018). Half an hour before observation, mice were transited to separated cages. Two items of undisturbed home cage observations and three handling observations were carried out 4 weeks after TAC by two observers blindly and independently, and the average scores were recorded. Score criteria were: 1) Locomotor activity in cage and resistance when gripped: normal = 0, decrease = −1, increase = 1. 2) Respiration: 5 = normal; 4 = slightly incomplete/fast and shallow, bradypnea; 3 = moderately incomplete/rapid breathing, difficulty breathing; 2 = severely incomplete/wheezing, breathing with mouth open; 1 = weak breathing. 3) Reactivity to grabbing: 5 = very difficult; 4 = difficult; 3 = somewhat difficult; 2 = easy/normal; 1 = very easy. A detailed description of the score criteria is in the Supplemental Materials. Grip strength (Bellantuono et al., 2020) was measured using a rats/mice grip strength meter (YSL-13A) according to the manufacturer’s instructions. The total distance was calculated from the open field test (Sun et al., 2021) in 5 min using Noldus Ethovision 3.1.

### RNAseq and RT-qPCR

Frozen heart tissues of 4-week post-TAC or sham C57BL/6J mice were sent to Novogene Technology Co., Ltd. (Beijing, China) for transcriptome sequencing. Among differentially expressed genes (q-value < 0.05), 15 genes of interest were further verified in ICR and C57BL/6J mice. Regarding validation, total RNA was isolated from heart tissues using Trizol reagent (Life Technologies), and cDNA was synthesized using Superscript Ⅲ reverse transcriptase and OligodT primer following the manufacturer’s instructions (Life Technologies). Additionally, real-time quantitative PCR was performed on the StepOnePlus system using SYBR green (Lablead) and primers listed in Supplementary Table S1. Ct values of targeted mRNA were normalized to *Gapdh*. Relative expressions of these genes were calculated by the 2^−ΔΔCT^ method.

### Statistics

Data analyses were conducted using GraphPad Prism 8.0, with a 2-tailed Student’s *t*-tests or one-way ANOVA followed by a post hoc Tukey-Kramer test. Furthermore, survival data were performed using Kaplan–Meier survival analysis with a log-rank statistical method. Finally, the heatmap of gene expression was presented based on normalized data. Quantitative data are presented as means ± SEM, and statistical significance was achieved when *p* ≤ 0.05.

## Results

### Overall survival

**FIGURE 1:**
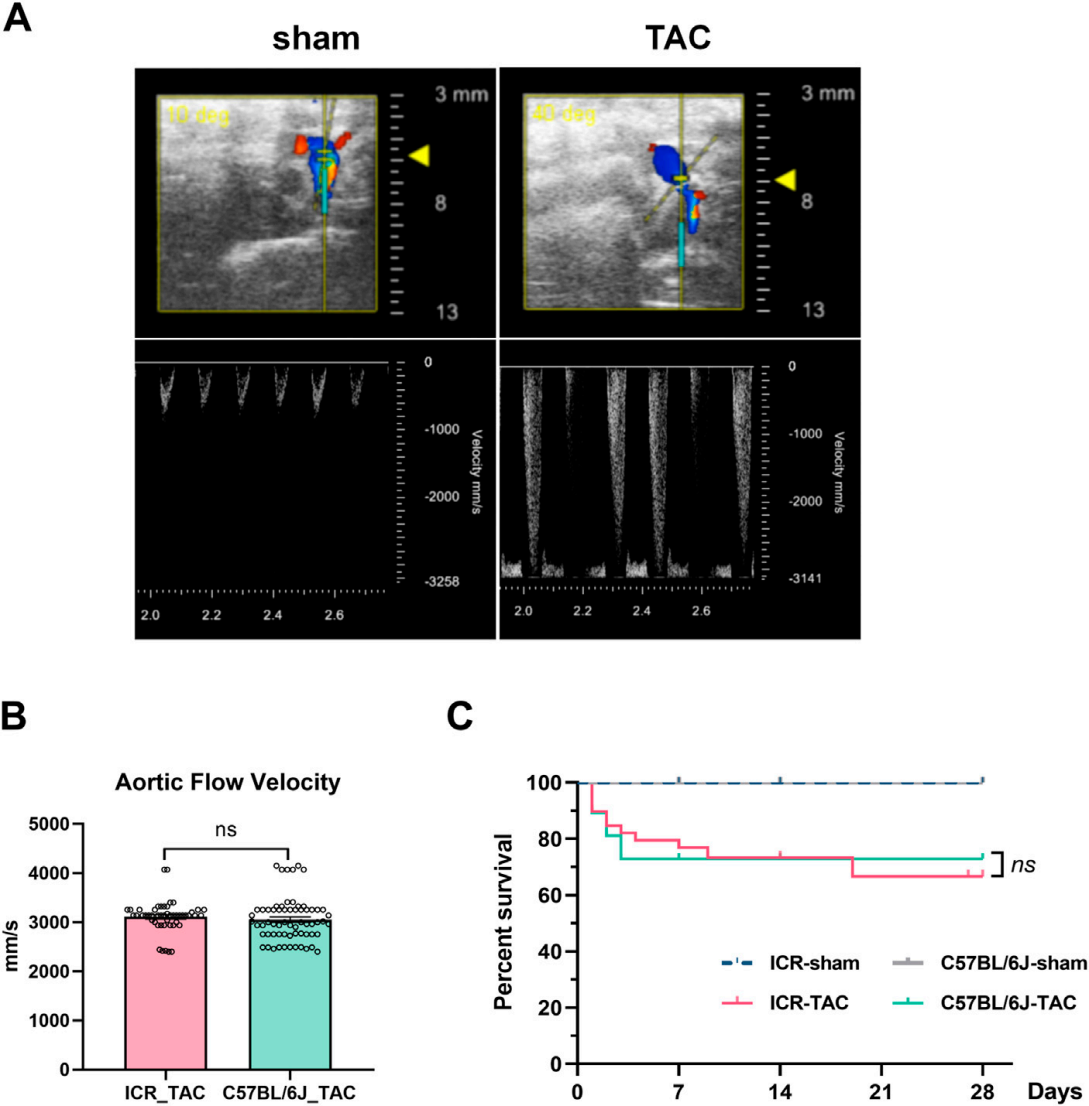
Survival after transverse aortic constriction (TAC) in ICR and C57BL/6J mice. **(A)** Representative Doppler flow velocity waveforms (lower panel) obtained from the aortic arch (upper panel) after TAC surgery in ICR and C57BL/6J mice. Inclusion criteria: Vel_sham_ ≤ 800 mm/s, Vel_TAC_ ≥ 2400 mm/s. **(B)** Aortic flow velocity after TAC surgery from ICR and C57BL/6J mice. **(C)** Kaplan-Meier survival curve analysis of ICR and C57BL/6J mice after TAC surgery. *N* = 21, 39 for sham and TAC group of ICR, 23, 37 for sham and TAC group of C57BL/6J, respectively. Ns: not significant.

To ensure successful aortic arch constriction, we performed the pulsed-wave doppler at the ligated site for each mouse. No constriction with normal aortic peak flow velocity (usually ≤800 mm/s) was observed in the sham-operated group. In contrast, flow velocity was significantly increased in the TAC group, indicating higher heart pressure (Figure 1A). Therefore, for intragroup homogeneousness, only when the flow velocity was ≥2400 mm/s could they be enrolled in the TAC group for further study. There was no difference in aortic flow velocity between ICR and C57BL/6J mice after TAC (Figure 1B). Mice that completed the surgical operation and recovered from anesthesia were monitored for Kaplan–Meier survival rate analysis for 4 weeks. Sham mice either in the ICR or C57BL/6J groups, all survived till the end of the study. However, 11 deaths were recorded in the ICR TAC group with the survival rate gradually declining to 64.52%, whereas 10 deaths occurred in the C57BL/6J group within 3 days post-TAC. Nonetheless, there was no significant difference in the overall survival till 4 weeks between both strains (Figure 1C).

### Echocardiography

**FIGURE 2:**
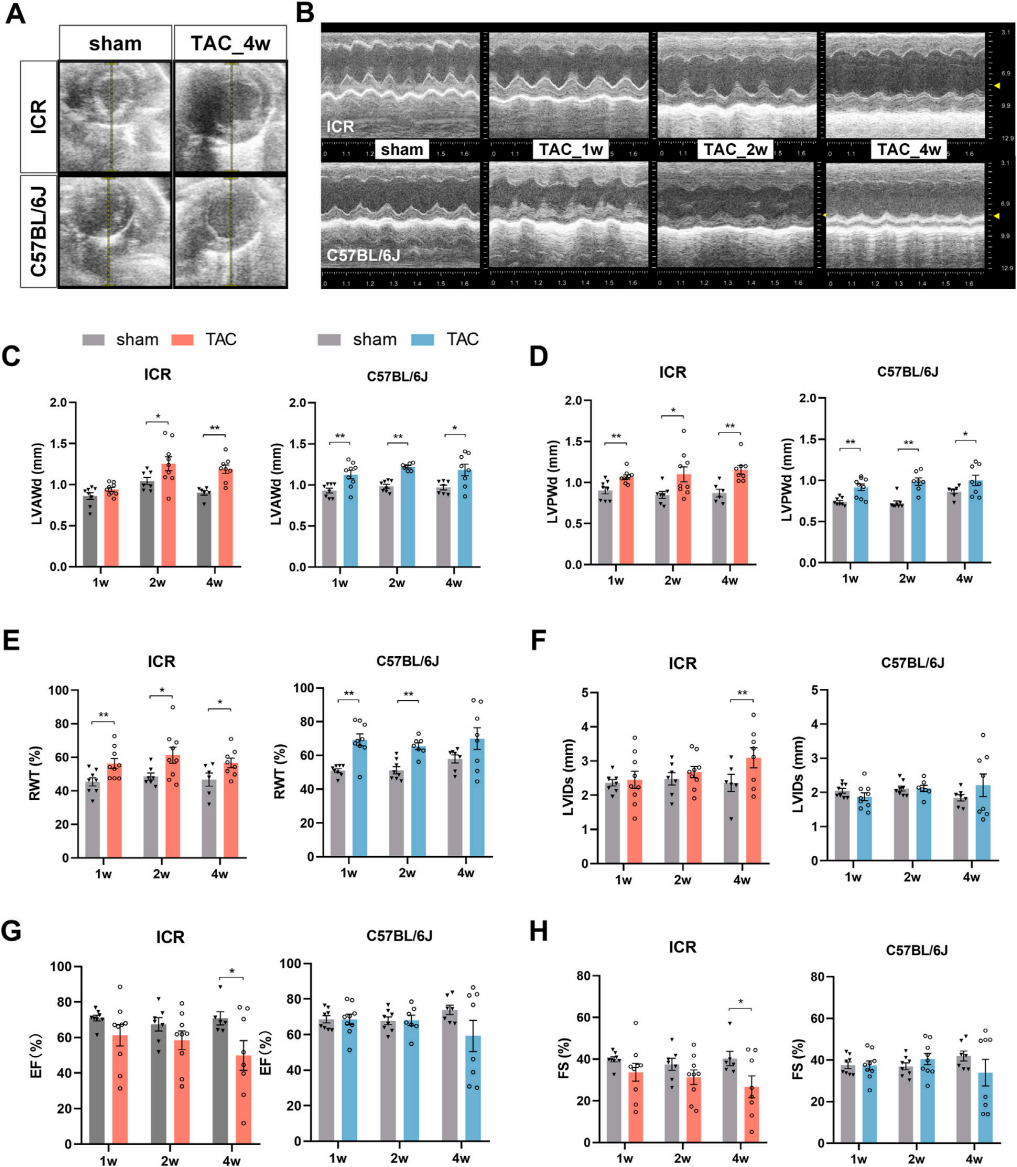
Echocardiography at 1, 2 and 4 weeks post-TAC or sham surgery in ICR and C57BL/6J mice. **(A,B)** Representative ultrasound images of left ventricle (short-axis, at the level of the papillary muscles) from ICR and C57BL/6J mice: **(A)** in B-mode; **(B)** M-mode. **(C–H)** Averaged parameters obtained from analysis of echocardiograms: **(C)** Left ventricular anterior wall thickness at end-diastole (LVAWd); **(D)** LV posterior wall thickness at end-diastole (LVPWd); **(E)** Relative Wall thickness (RWT) = (LVAWd + LVPWd)/LVIDd×100%. **(F)** LVID at end systole (LVIDs); **(G)** Ejection fraction (EF); **(H)** Fractional shortening (FS); Values represent mean ± SEM, *N* = 6–9 per ICR group,*N* = 7–9 per C57BL/6J group. *p < 0.05, **p < 0.01.

We assessed the cardiac structure and pump function in living animals using echocardiography. A series of echocardiographic tests were conducted at one, two, and four weeks post TAC or sham surgery (Figures 2A,B). In the C57BL/6J mice, LVAWd and LVPWd increased significantly in week 1, peaked at week 2, then remained relatively stable till week 4 compared to the sham controls (Figures 2C,D). In the ICR mice, TAC also led to a significant increase in LVPWd from week 1 and in LVAWd from week 2 (Figures 2C,D). Consistent with the early thickened wall, C57BL/6J mice revealed significantly decreased LVIDd and LV volume 1 week post-TAC, indicating concentric remodeling at the beginning of the hypertrophic stimulation. This alteration recovered at week 2 but varied at week 4 without a significant difference (Supplementary Table S2). ICR TAC mice displayed a similar slight declining trend at week 1 but progressively recovered at week 4 in internal dimension and volume (Supplementary Table S2). Relative wall thickness (RWT) is a measure of LV geometry that the increase implies concentric remodeling or hypertrophy whereas its decrease indicates eccentric hypertrophy (Støylen et al., 2016). Figure 2E reveals that RWT in ICR and C57BL/6J TAC group increased significantly in week 1 and remained high until week 4 compared to each strain’s sham group. Notably, there was no statistical difference between the sham and TAC group in C57BL/6J at week 4, perhaps due to individual variation.

Combined with the end-systole parameters, the systolic function can be calculated. In ICR TAC mice, the LVIDs and LV volumes rose time-dependently whereas fractional shortening (FS) and ejection fraction (EF) displayed a continuous fall trend; therefore, all attained significance at week 4 (Figures 2F–H and Supplementary Table S2). In C57BL/6J TAC mice, those parameters remained steady in the first 2 weeks compared to the sham group, whereas the same trends were observed until week 4 without statistical significance. Despite being observed in both strains, phenotyping heterogeneity was more significant in C57BL/6J at the endpoint. Therefore, half experienced reduced FS and EF while the other half remained conserved or increased (Figures 2G,H).

### Heart and lung weight

**FIGURE 3:**
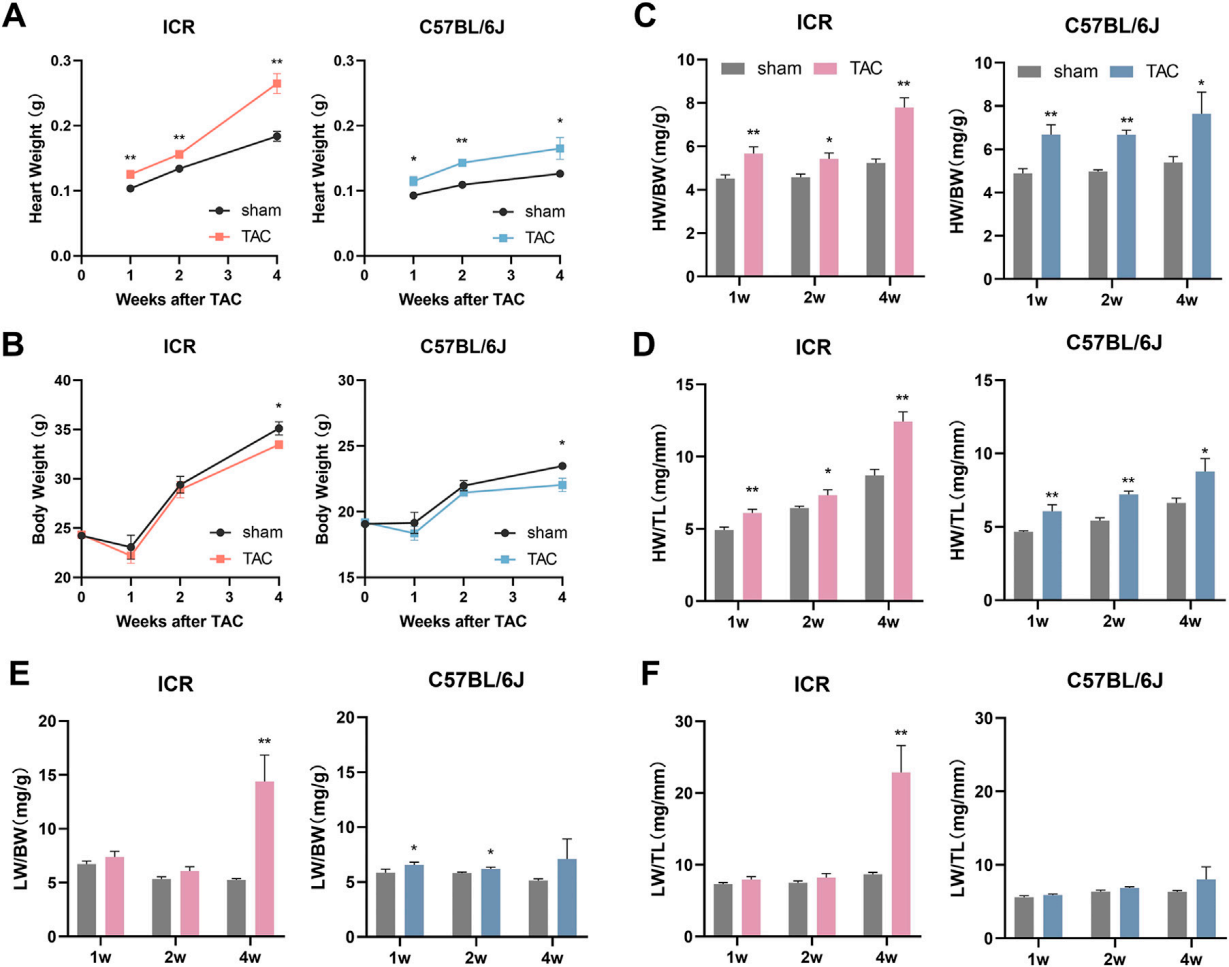
Statistical results of Organ coefficients at 1, 2 and 4 weeks post-TAC surgery or sham in ICR and C57BL/6J mice. **(A)** Heart weight. **(B)** Body weight. **(C,D)** Heart weight normalized to body weight (HW/BW) or tibia length (HW/TL). **(E,F)** Lung weight normalized to body weight (LW/BW) or tibia length (LW/TL). Data are shown as mean ± SEM, *N* = 6–8 per ICR group, *N* = 6–9 per C57BL/6J group. *p < 0.05, **p < 0.01 vs. their specific sham group.

The heart and lung are the most susceptible organs to cardiovascular diseases. Consistent with echocardiographic data of LV structural changes, both strains of mice demonstrated a remarkable and sustained increase in heart weight (HW) in response to TAC during the 4-week observation period (Figure 3A). By normalizing body weight (BW) or tibia length (TL), the changes persisted significantly (Figures 3C,D). Unlike HW/TL, HW/BW in the initial 2 weeks did not keep rising, which is attributable to the sharp fall in BW 1-week postoperatively (Figure 3B). The wet lung weight (LW) normalizing to BW was marginally increased in C57BL/6J mice within the first 2 weeks post-TAC while LW/TL change was not significant enough (Figures 3E,F). This minor inconsistency between two lung indices could also be attributable to decreased BW but stable TL. At week 4, the increases of LW/BW and LW/TL in C57BL/6J TAC did not achieve significant difference yet (Figures 3E,F). Quite different in ICR mice, LW/BW and LW/TL scarcely changed in the initial 2 weeks post TAC but elevated dramatically by 179.33% and 165.05%, respectively, at week 4 (Figures 3E,F), indicating severe pulmonary congestion due to cardiac dysfunction.

### Morphologic and histologic analysis

**FIGURE 4:**
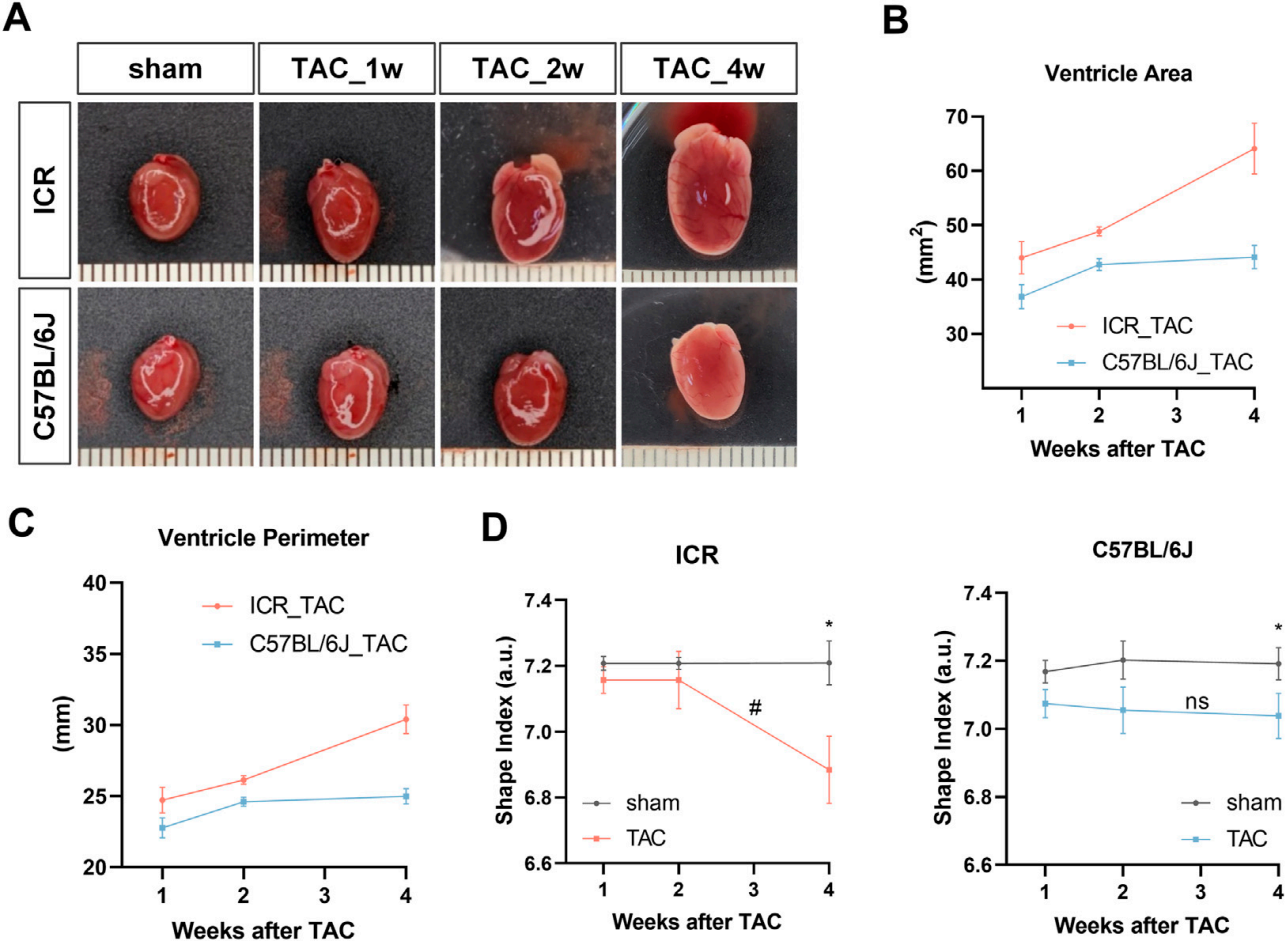
Gross samples and shape analyses of mouse hearts at 1, 2 and 4 weeks post-TAC or sham surgery in ICR and C57BL/6J mice. **(A)** Representative photos of whole heart. Scale bar is 1 mm. **(B)** Ventricle area (left and right ventricle) and **(C)** ventricle perimeter obtained from heart photos. **(D)** Shape index (area over perimeter squared). Data are shown as mean ± SEM, *N* = 6 per ICR group, *N* = 6–8 per C57BL/6J group. *p < 0.05, **p < 0.01 vs. their specific sham group. ^#^ *p* < 0.05, ns denotes not significant compared between 2w and 4w in TAC group.

In agreement with the heart weight data, both strains of mice had gradually expanded shapes in the major axis and minor axes, evidenced by the gross heart photos following TAC (Figure 4A). To quantitatively assess the shape alteration, the shape index was calculated based on ventricle area and perimeter (Dvornikov et al., 2019), measured from each heart photo. The line chart showing that ventricle area and perimeter in ICR TAC mice grew continuously with steep slopes, whereas in C57BL/6J mice, these parameters grew fast from week 1 to week 2 but mildly till week 4 (Figures 4B,C). Figure 4D illustrates a stable shape index (7.21 for ICR, 7.17–7.20 for C57BL/6J) in all the sham groups, indicating a rounder shape. In ICR TAC mice, the shape index had no significant changes within 2 weeks but fell sharply by 4.50% compared to sham at week 4, which was also significantly lower than week 2. However, in C57BL/6J TAC mice, the shape index declined almost significantly (by 1.31%, *p* = 0.053) from week 1 but continued mildly till week 4 (by 2.09%, *p* < 0.05) compared to their sham controls (Figure 4D). Together, these data suggest cardiac remodeling development in ICR is much more severe in response to LV pressure overload for 4 weeks. In contrast, it is earlier and milder in C57BL/6J strains from a flat two-dimensional surface perspective.

**FIGURE 5:**
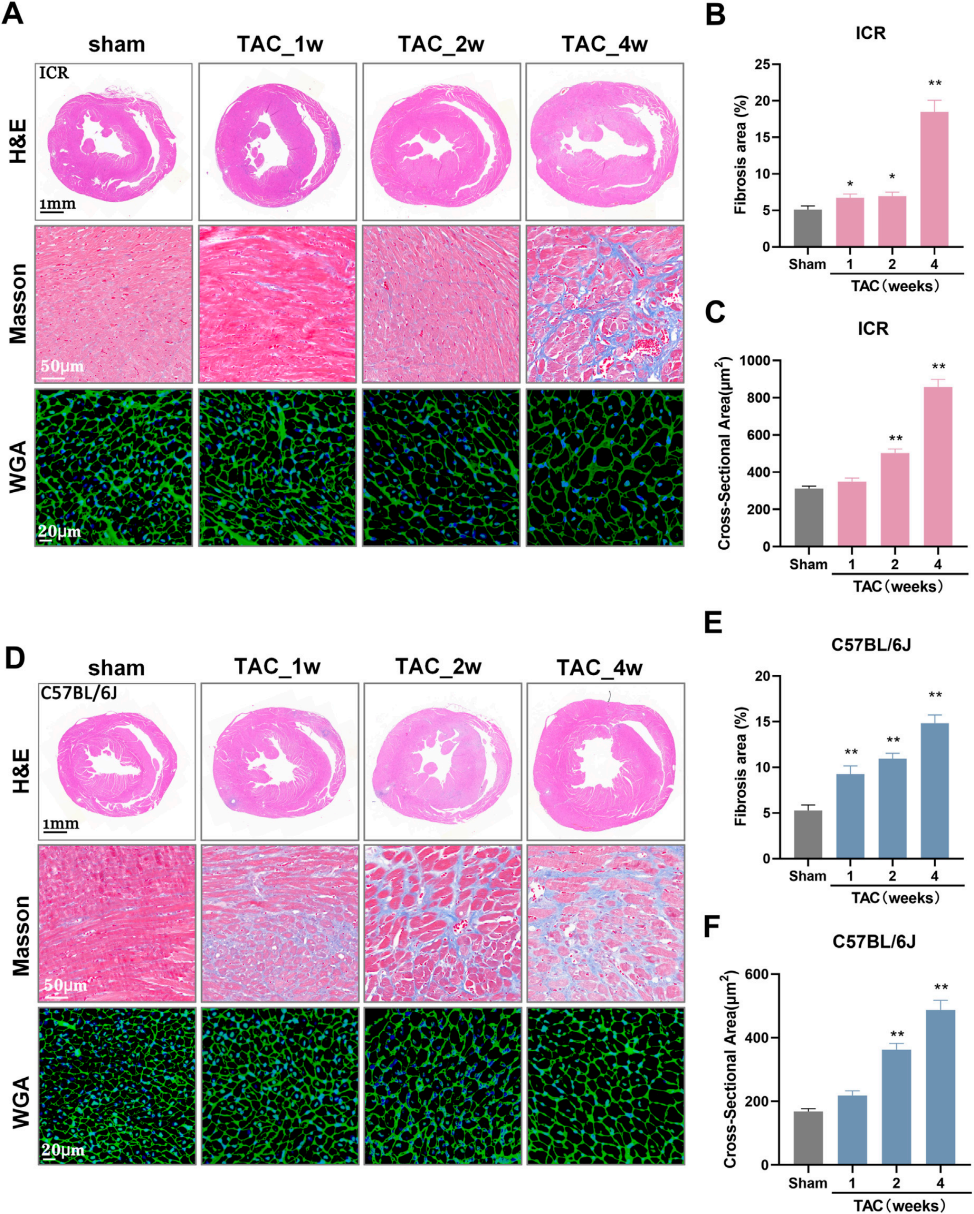
Histological alterations of mouse hearts at 1, 2, and 4 weeks post-TAC surgery in ICR and C57BL/6J mice. **(A)** Representative images of H&E-stained heart sections (upper panel), scale bar = 1 mm; Masson trichrome-stained left ventricle tissues (middle panel), red stain denotes muscle fibers while blue stain denotes collagen. White scale represents 50 μm; Wheat-germ-agglutinin (WGA)-stained cardiomyocytes border (lower panel), scale bar = 20 µm. All images obtained from ICR mice. **(B)** Quantitative analysis of interstitial fibrotic area measured from Masson-stained pictures and **(C)** Cardiomyocyte cross-sectional area measured from WGA-stained pictures in ICR mice. **(D–F)** The same indicators of **(A–C)** in C57BL/6J mice. *N* = 3 per group per staining. Quantitative data are shown as mean ± SEM, **p < 0.01 vs. sham.

Histological heart sections revealed wall thickening with LV dilation, enlarged cardiomyocyte size and extensive interstitial fibrosis, progressively in both strains following TAC (Figures 5A,D). By quantifying the blue-stained area ratio, we observed that the fibrosis area in the sham group is approximately 5%, while TAC-induced fibrosis growth was detected in week 1. The fibrosis area increased gradually to 14.83% at week 4 in C57BL/6J TAC mice, whereas in ICR TAC models it remained at 6.73% and 6.94% at week 1 and 2, respectively, but soared to 18.49% at the end of the study (Figures 5B,E). Regarding the cross-sectional area of individual cardiomyocytes, both strains revealed similar growth patterns with significance initially attained at 2 weeks post-TAC and almost thrice larger than sham controls at week 4 (Figures 5C,F). ICR mice recapitulates the main histopathological characters as C57BL/6J developed response to TAC.

### Behavioral tests

**FIGURE 6:**
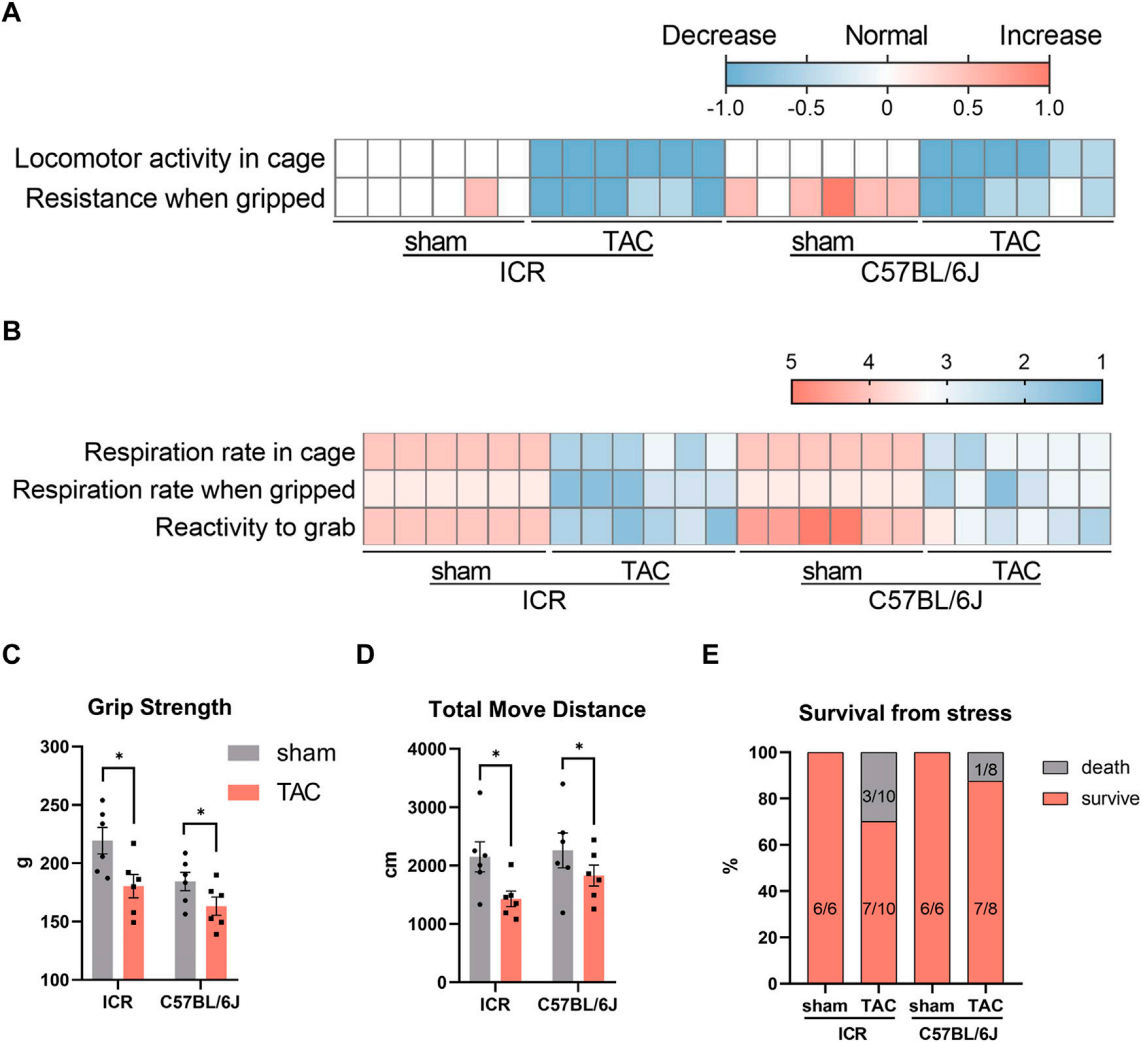
Behavior observation at 4 weeks after TAC in ICR and C57BL/6J mice. **(A)** Heatmap of locomotor activity in cage and resistance when gripped. Score: normal = 0, decrease = −1, increase = 1. **(B)** Respiration rate in cage or gripped and reactivity to grab. Score of respiration: 5 = normal; 4 = slightly incomplete/fast and shallow, bradypnea (breathing either fast and shallow or slow); 3 = moderately incomplete/rapid breathing, difficulty breathing (breathing very fast and shallow or very shallow and laboured in appearance); 2 = severely incomplete/wheezing, breathing with mouth open (wheezing or breathing with mouth open); 1 = weak breathing (breathing very little). Score of reactivity to grab: 5 = very easy (sits and allows itself to be picked up); 4 = easy/normal (does not resist); 3 = somewhat difficult (stands); 2 = difficult (cringes and becomes rigid or runs around and is difficult to grab); 1 = very difficult (attacks). **(C)** Grip strength measured by a rats/mice grip strength meter. **(D)** Total move distance calculated from open field observation in 5 min. *N* = 6 each in **(A–D)**, quantitative data are shown as mean ± SEM, *p < 0.05. **(E)** Percent survival from stress like anesthesia, hair removal, echocardiographic test.

Animal behavioral tests are essential to neurological and psychological studies, which have gradually extended to other fields, including cardiology, like the swimming capacity of zebrafish (Kim et al., 2021) and spontaneous activity by open field test (OPT) of rodents (Sun et al., 2021). Therefore, we subsequently conducted behavioral tests to evaluate how the heart remodeling impacted the physical ability 4 weeks post- TAC. First, locomotor activity and respiration rate within the cage were assessed using undisturbed home cage observations. Compared to normal locomotor activity in the sham groups, all TAC mice displayed less spontaneous activity (Figure 6A). Furthermore, the respiration rate in sham mice was normal within the cage; however, it got fast and shallow when gripped in hand. Contrastingly, TAC mice demonstrated moderate or severe incomplete breathing within the cage, which worsened when they were gripped compared to the sham group (Figure 6B). In addition, the sham C57BL/6J mice were more challenging to catch and struggled fiercely when griped compared to the ICR mice, whereas in the TAC group, all the mice were much easier to grab and hardly struggled evidenced by their lower scores (Figure 6B). Notably, activity decreased, and breath condition seemed more severer in the ICR TAC group than in the C57BL/6J group. Similarly, in the grip strength test and OPT, TAC mice of both strains displayed decreased strength and significantly shorter moving distances compared to the sham mice, probably owing to a much weaker condition (Figures 6C,D). Additional 3 of 8 mice died after echocardiography or indispensable preparations before the test, 1 of 7 for the C57BL/6J mice and none of the shams (Figure 6E). Therefore, these data suggest that the C57BL/6J strain is more tolerant than the ICR regarding response to pressure overload for 4 weeks.

### Genes expression analysis

**FIGURE 7:**
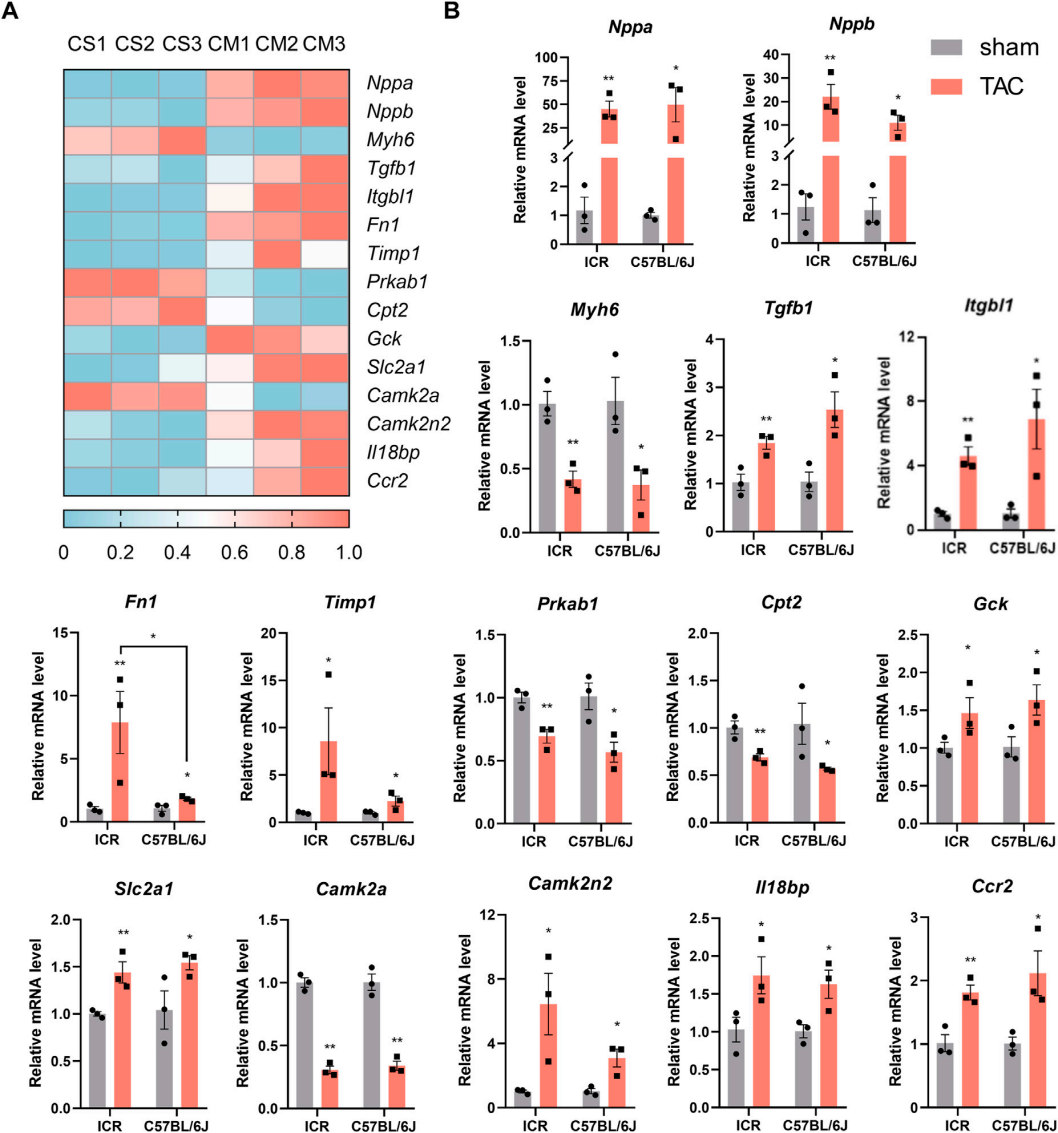
Pathological gene-expression alteration in heart tissue of ICR and C57BL/6J mice 4 weeks post-TAC. **(A)** Heatmap of differentially expressed genes in C57BL/6J mice by RNAseq. CS: C57BL/6J sham; CM: C57BL/6J model. *N* = 3. **(B)** Real time quantitive PCR verification of the expression levels of 15 selected genes in both ICR and C57BL/6J mice. Relative mRNA levels are normalized to *Gapdh*, values are shown as mean ± SEM, *N* = 3 each. *p < 0.05, **p < 0.01 vs. their own sham.

In mice that survived until 4 weeks post-TAC, we performed RNA-seq using heart tissues of the C57BL/6J strain and observed the expected gene expression patterns of major pathological change markers. In addition, the heatmap illustrated 15 out of thousands of differentially expressed genes involved in hypertrophy, fibrosis, energy remodeling, calcium handling, and inflammatory reaction (Figure 7A). Next, we validated those genes’ expression in both strains using qPCR. As expected, the expression of natriuretic peptides A (*Nppa*) and natriuretic peptides B (*Nppb*) rose by about 40 and 20 times of the sham controls. In contrast, myosin heavy polypeptide 6 (*Myh6*) was significantly downregulated by approximately 60% in both strains post-TAC (Figure 7B). Genes involved in fibrotic remodeling were vigorously expressed, evidenced by elevated *Tgfb1*, *Itgbl1*, *Fn1,* and *Timp1* in TAC hearts. The lower levels of *Prkab1* and *Cpt2* and higher expressed *Gck* and *Slc2a1* implied energy deficiency due to insufficient fatty acid oxidation while being replaced with higher glucose utilization. TAC-induced disorder of calcium signaling and inflammatory response were validated by downregulation of *Camk2a* and upregulation of *Camk2n2*, *Il18bp,* and *Ccr2* in both strains (Figure 7B). To further explore the differences in expression of genes between the two TAC models, we found the elevated *Fn1* was significant higher in ICR (7.88-fold) than C57BL/6J (1.82- fold). Overall, the ICR TAC model demonstrated similar pathological gene expressions as C57BL/6J which could to be applied to pathophysiological and pharmacological investigations.

## Discussion

In the present study, we conducted a comprehensive evaluation of TAC models using ICR and C57BL/6J mice. We observed that the ICR strain developed cardiac hypertrophy after 2 weeks of TAC exposure, evidenced by increased wall thickness, heart weight, and enlarged cardiomyocyte size compared to sham controls during the same period. The C57BL/6J TAC mice developed significant concentric hypertrophy at week 2 or earlier; however, the systolic function was preserved with higher variability throughout the 4 weeks of pressure overload. Conversely, the ICR strain was sensitive to pressure overload-induced HF supported by reduced EF, severe lung congestion, rapid progressive fibrotic remodeling with higher *Fn1* expression, poor behavioral performance and survival from stress compared to the C57BL/6J mice. Besides, major gene expression pattern responses to TAC in C57BL/6J mice can be validated in ICR strain.

From our own experience, two technical points of TAC surgery should be emphasized to avoid unexpected death: 1) The perforation between the aortic arch and connective tissue should be cautiously done; otherwise, instantaneous death would happen due to collapsed lungs since there is no ventilator support. 2) The exact constricted position should be between both carotid arteries to ensure that no carotid artery is ligated together with the aorta; otherwise, the animal would develop lethal cerebral ischemia with complicated motor disorders.

Mice subjected to TAC exhibit adverse effects of cardiac remodeling of various severity (Mohammed et al., 2012), potentially influenced by multiple study characteristics like genetic background, sex, degree of constriction, follow-up time, and others factors. To optimize the TAC model design, we controlled several factors. Specifically, they were male with comparable body weight, anesthetized with phenobarbital *via* intraperitoneal injection. They underwent minimally invasive TAC surgery using a 27-gauge needle adapter for constriction, which was preferred by the majority (Bosch et al., 2021). Study follow-up time ranged from 1.5 days (Suetomi et al., 2018) to 280 days (Dzhonova et al., 2018), with most commonly 28 days (Bosch et al., 2021) depending on the study purpose and we aimed to determine how long mice developed phenotypes of HF. The current study ended 4 weeks post TAC when ICR mice were detected with remarkable cardiac function deterioration and lung congestion.

Developing a reliable TAC-induced HF model is crucial to investigating the underlying mechanisms and pharmacological intervention of pressure overload-induced HF. A concern gradually raised by the C57BL/6J TAC model is the HF-related phenotype variation. Researchers claimed the C57BL/6J strain is resistant to chronic pressure overload that hardly transited to HF for a considerable period (Mohammed et al., 2012; Garcia-Menendez et al., 2013; Zi et al., 2019; Tannu et al., 2020). Therefore, a consecutive study that enrolled 43 mice revealed that 72% of mice subjected to TAC with a 27G constriction did not develop heart failure even at 9 weeks postoperatively (Mohammed et al., 2012). A similar study revealed that 75% subpopulation of C57BL/6J mice did not develop HF until 5 weeks following TAC (Zi et al., 2019). Consistent with those findings, half the C57BL/6J mice sustained preserved systolic function the following 4 weeks after TAC. Theoretically, the constriction degree accelerates the HF transition; however, thinner banding adapters inevitably cause greater mortality (Zi et al., 2019). Additionally, we observed that mortality in the C57BL/6J TAC mice were all happened within 3 days, attributable to poor tolerance to drastically changed conditions postoperatively. Therefore, increasing constriction may not be a good strategy for C57BL/6J mice. Usually, high variability within the same group affects data interpretation and conclusion, and more samples are required to achieve significant differences. As a recent study introduced, TAC-induced HF models experienced 3 periods: ventricular hypertrophy, heart failure with preserved EF (HFpEF), and heart failure with reduced EF (HFrEF) corresponding to 2w, 4w and 8w post a 26G construction in 3-month old C57BL/6J mice (Li et al., 2022). Though quite different from our experimental settings, the insight is valuable. Thus, the C57BL/6J strains is an ideal model for studying cardiac hypertrophy and HFpEF. In contrast, for ICR, the relatively low variability and robust cardiopulmonary phenotypes at 4 weeks post-TAC make this strain more suitable as a HF model.

There is a noticeable change that a rapid progress of pathological phenotypes happened between week 2 to week 4 post-TAC in ICR mice. We tried to explain this phenomenon from three points. First, sustained weight gain increases the extra workload on the heart. It is well accepted that obesity is strongly independently related to cardiovascular disease development and correlative mortality than any other risk factors (Powell-Wiley et al., 2021). Furthermore, most obesity-related investigations are conducted based on ICR background (Liu et al., 2017; Jiang et al., 2020). A recent study compared four strains including C57BL/6J to assess obesity development sensitivity in response to high-fat diets and revealed that ICR gained the most weight within 10 weeks in normal and high-fat diets (Li et al., 2020), indicating an “obesity easy” trait. From our data, 10.88 and 9.45 g were gained in ICR sham and TAC, respectively, and 4.41g and 2.85g in the C57BL/6J mice during the 4 weeks. The endpoint body weight of the ICR model was 1.5 times that of C57BL/6J. The criteria for mice obesity are unclear; nonetheless, a higher body weight infers higher blood supply demand, increasing the strain on the heart. Secondly, it is possible that the percentage of constriction degree increased over time in ICR mice. To make a comparable reduction of aortic flow to approximately 70%, Tannu et al. used a 0.38-mm and 0.41-mm banding adapter for BALB/c and C57BL/6J mice, respectively, since the former demonstrated a smaller aortic diameter at baseline (Tannu et al., 2020). Unfortunately, we did not measure the aortic diameter pre- and post-TAC, which might widen as mice gain weight. If ICR has a growing aortic diameter with age, we postulated that the fixed 27-gauge (0.41 mm) ligation could bring increased pressure to the heart due to greater constriction than before. Thus, the extra workload from significant weight gain and possible growing constriction degree promoted the rapid transition of ICR mice from hypertrophy to heart failure in the last 2 weeks. Another interest finding is that fibrosis area as well as *Fn1* gene expression were higher in ICR TAC model than C57BL/6J ones. Cellular fibronectin (FN), encoded by *Fn1*, is a multifunctional glycoprotein which secreted by several cell types into extracellular matrix in the heart. FN levels are increased in HF animal models and inhibiting FN attenuates fibrosis and improves cardiac function (Valiente-Alandi et al., 2018). The prominent upregulation of *Fn1* gene is presumed to accelerate HF progression in ICR TAC model.

Furthermore, we would like to emphasize two points based on this study. One is the importance of setting sham controls at each parallel time point, as many crucial indices varied with age even in the sham group. For future pharmacological studies, a good *in vivo* model should distinguish normal control and be sensitive enough to reveal the drug efficacy. Therefore, the study design should consider a simultaneous comparison with the parallel sham control. The other points is that organ weights corrected to tibia length but not body weight is more reliable when body weight may affected by some experimental conditions like surgery.

Our study also had some limitations. We did not measure cardiac structure and function before TAC and assumed all the mice had an inconsiderable variance of these indices at baseline. Furthermore, an extended follow-up period after TAC till the model mice display HF phenotypes is preferable, and more enrolled samples might help reduce variance. We compared the pathologiocal gene expression patterns between the two mice strains while further validation in protein translation level would be benefical.

In summary, this study provides fundamental information for further studies using ICR mice to build a TAC model and study pressure overload-induced hypertrophy and heart failure. In addition, ICR mice demonstrated clinically relevant heart failure profiles post-TAC, including progressive hypertrophic remodeling, massive fibrosis, and reduced systolic function with serious pulmonary congestion, which can be a good research tool to investigate potential drugs and targets for pressure overload-induced HF.

## Data Availability

The datasets presented in this study can be found in online repositories. The names of the repository/repositories and accession number(s) can be found in the article/Supplementary Material.
