# Supplementary material for: Evaluation of the transverse aortic constriction model in ICR and C57BL/6J mice
Source: Front Physiol. 2022 Nov 29;13:1026884. doi: 10.3389/fphys.2022.1026884 (PMC9745147; doi:10.3389/fphys.2022.1026884)
Supplement: Supplementary file 1 [file Table1.docx]

Supplementary Material

**Supplementary Table 1. Primers for qPCR**

| **Gene** | **Forward** | **Reverse** |
| --- | --- | --- |
| ***Nppa*** | ATCCTGTGTACAGTGCGGTG | TACCGGCATCTTCTCCTCCA |
| ***Nppb*** | GAAGGACCAAGGCCTCACAA | ACTTCAGTGCGTTACAGCCC |
| ***Myh6*** | TCTGCCTACCTTATGGGGCT | ACTTGCTGTACACTCTGCCC |
| ***Tgfb1*** | ACTGGAGTTGTACGGCAGTG | GGGGCTGATCCCGTTGATTT |
| ***Itgbl1*** | CTGGTTGGCATGGCGATAAA | GATGTGCATCTTCGCTTGCT |
| ***Fn1*** | ATGAGAAGCCTGGATCCCCT | GGAAGGGTAACCAGTTGGGG |
| ***Timp1*** | CCCCAGAAATCAACGAGACCA | ACTCTTCACTGCGGTTCTGG |
| ***Prkab1*** | CCTTTCCGGCTTCCTTGTGT | AAGATGTCGGCGTCTTCAGG |
| ***Cpt2*** | TGTGAGCGGAAGATCCCAAC | GCTTTCCAACCCGATCTCCT |
| ***Gck*** | TTGCAACACTCAGCCAGACA | GGGCTCCCCTCCTTGTAGTA |
| ***Slc2a1*** | TACACCCCAGAACCAATGGC | CCCGTAGCTCAGATCGTCAC |
| ***Camk2a*** | CCCCAAAGTCACAGAGCCAT | ACGTGTCGTCTTCCACTGTC |
| ***Camk2n2*** | CCTACCCTACGGTGAGGACA | CCAGCGAAGAAGGAGTTGGT |
| ***Il18bp*** | TTAGGAGCCAGAAGGAGGCT | ACAAAAGCAGGACCCACCAA |
| ***Ccr2*** | GCCATCATAAAGGAGCCATACC | ATGCCGTGGATGAACTGAGG |
| ***Gapdh*** | CCCTTAAGAGGGATGCTGCC | TACGGCCAAATCCGTTCACA |

**Supplementary Table 2. Echocardiography parameters**

| **Index** | **Strain** | **1w** | | **2w** | | **4w** | |
| --- | --- | --- | --- | --- | --- | --- | --- |
|  |  | **sham** | **TAC** | **sham** | **TAC** | **sham** | **TAC** |
| **LVAWs (mm)** | **ICR** | 1.26 ± 0.06 | 1.34 ± 0.05 | 1.51 ± 0.05 | 1.70 ± 0.09* | 1.44 ± 0.08 | 1.62 ± 0.05* |
|  | **C57BL/6J** | 1.37 ± 0.06 | 1.52 ± 0.08 | 1.50 ± 0.05 | 1.70 ± 0.05** | 1.33 ± 0.04 | 1.58 ± 0.10* |
| **LVPWs (mm)** | **ICR** | 1.37 ± 0.05 | 1.44 ± 0.06 | 1.35 ± 0.08 | 1.47 ± 0.08 | 1.31 ± 0.06 | 1.52 ± 0.07* |
|  | **C57BL/6J** | 1.08 ± 0.03 | 1.33 ± 0.07** | 1.08 ± 0.05 | 1.37 ± 0.05** | 1.39 ± 0.06 | 1.36 ± 0.13 |
| **LVIDd（mm）** | **ICR** | 3.93 ± 0.31 | 3.63 ± 0.48 | 3.94 ± 0.38 | 3.89 ± 0.28 | 3.90 ± 0.64 | 4.18 ± 0.43 |
|  | **C57BL/6J** | 3.28 ± 0.13 | 2.97 ± 0.26** | 3.35 ± 0.24 | 3.37 ± 0.15 | 3.17 ± 0.21 | 3.23 ± 0.55 |
| **LV Mass AW (Corrected) (mg)** | **ICR** | 104.56 ± 5.57 | 111.73 ± 7.76 | 116.11 ± 6.80 | 157.58 ± 13.61* | 104.53 ± 8.90 | 167.89 ± 14.42** |
|  | **C57BL/6J** | 72.67 ± 2.69 | 84.56 ± 5.38* | 77.55 ± 2.70 | 113.39 ± 2.93** | 79.71 ± 1.67 | 103.16 ± 8.43* |
| **LV Vol;d (uL)** | **ICR** | 67.47 ± 4.40 | 56.96 ± 5.91 | 68.14 ± 5.64 | 65.82 ± 3.79 | 68.20 ± 10.67 | 78.91 ± 6.76 |
|  | **C57BL/6J** | 43.51 ± 1.48 | 34.57 ± 2.49** | 46.22 ± 2.76 | 46.72 ± 1.86 | 40.36 ± 2.43 | 42.88 ± 6.95 |
| **LV Vol;s (uL)** | **ICR** | 19.76 ± 1.94 | 24.35 ± 5.79 | 23.03 ± 3.88 | 27.79 ± 4.14 | 21.13 ± 4.65 | 41.71 ± 8.87* |
|  | **C57BL/6J** | 13.74 ± 1.21 | 11.37 ± 1.87 | 14.86 ± 1.37 | 14.98 ± 1.55 | 10.60 ± 1.33 | 21.10 ± 6.94 |

* P<0.05, **P<0.01 compared with sham at same timepoint. N = 6-9 per ICR group，N = 7-9 per C57BL/6J group

**Score criteria of bahavior test**

1. **Locomotor activity in cage**

normal = 0, decrease = -1, increase = 1.

1. **Resistance when gripped**

normal = 0, decrease = -1, increase = 1.

1. **Respiration:**

5 = normal;

4 = slightly incomplete/fast and shallow, bradypnea (breathing either fast and shallow or slow);

3 = moderately incomplete/rapid breathing, difficulty breathing (breathing very fast and shallow or very shallow and labored in appearance);

2 = severely incomplete/wheezing, breathing with mouth open (wheezing or breathing with mouth open);

1 = weak breathing (breathing very little).

1. **Reactivity to grab:**

5 = very difficult (attacks).

4 = difficult (cringes and becomes rigid or runs around and is difficult to grab);

3 = somewhat difficult (stands);

2 = easy/normal (does not resist);

1 = very easy (sits and allows itself to be picked up);

**download link for original source data**

https://www.jianguoyun.com/p/DfbIwpEQ4MbvChjhjtQEIAA
